# Teasing apart the cell death pathways in HIV pathogenesis

**DOI:** 10.1038/s42003-021-02077-2

**Published:** 2021-04-21

**Authors:** Karli Montague-Cardoso

**Affiliations:** Communications Biology, https://www.nature.com/commsbio

## Abstract

The progressive loss of CD4 + T cells has been recognised as being central to HIV-1 pathogenesis, however a precise understanding of the underlying mechanisms and, consequently, improved therapies have yet to be achieved. Zhang et al. have recently shown in HIV-1 patients that the NLRP3 inflammasome pathway, which plays a key role in innate immunity, is a crucial mediator of the loss of CD4 + T cells. This advance could inform the development of innovative anti-HIV-1 therapies.

One of characteristics of chronic HIV-1 infection in patients is a progressive loss of CD4 + T cells, which consequently disrupts immune responses. A better understanding of the processes that mediate such depletion could pave the way for the development of more targeted anti-HIV therapies.

In a recent study, Zhang et al.^1^ demonstrated in viremic HIV-1–infected patients that a specific type of cell death—pyroptosis—plays a pivotal role in CD4 + T cell loss. Specifically, using peripheral blood samples from viremic patients as well as healthy controls, they used flow cytometry to monitor activation of both caspase-1 and caspase-3 in T cells, which are markers of pyroptosis and apoptosis, respectively. They found that pyroptotic and apoptotic CD4 + T cells constituted distinct populations with different phenotypes. In addition they observed that levels of both pyroptosis and apoptosis in CD4 + T cells were significantly elevated during HIV-1 infection, and were decreased following antiretroviral therapy.

**Figure:**
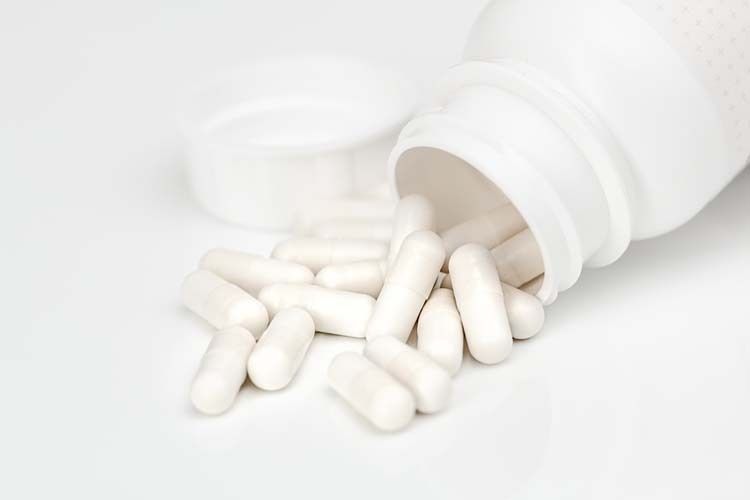
Pixabay

Critically, they also showed that pyroptosis was the more dominant pathway. Whilst current treatments can reduce the occurrence of pyroptosis, they cannot fully eradicate it. Zhang et al.^1^ also found that the mechanisms underlying depletion were different for pyroptotic and apoptotic CD4 + T cells. In the case of pyroptotic cells, they found that caspase 1activation was closely correlated with expression of inflammatory markers, such as cytokines, and was dependent on NLRP3 inflammasome activation, which plays a key role in innate immunity. In the case of the apoptosis however, caspase-3 activation in CD4 + T cells was more closely related to the T cell activation status.

Taken together, this study reveals that NLRP3 inflammasome-dependent pyroptosis plays a key role in CD4 + T cell loss in patients with HIV-1 infections. This could open up new avenues in the development of anti-HIV-1 therapies that target components of the pyroptosis pathway.
